# Current applications of adipose-derived mesenchymal stem cells in bone repair and regeneration: A review of cell experiments, animal models, and clinical trials

**DOI:** 10.3389/fbioe.2022.942128

**Published:** 2022-09-07

**Authors:** Zhengyue Zhang, Xiao Yang, Xiankun Cao, An Qin, Jie Zhao

**Affiliations:** ^1^ Shanghai Key Laboratory of Orthopedic Implants, Department of Orthopedics, Ninth People’s Hospital, Shanghai, China; ^2^ Shanghai Jiaotong University School of Medicine, Shanghai, China

**Keywords:** bone regeneration, osteogenesis, ASC, cell experiment, animal model, clinical trial

## Abstract

In the field of orthopaedics, bone defects caused by severe trauma, infection, tumor resection, and skeletal abnormalities are very common. However, due to the lengthy and painful process of related surgery, people intend to shorten the recovery period and reduce the risk of rejection; as a result, more attention is being paid to bone regeneration with mesenchymal stromal cells, one of which is the adipose-derived mesenchymal stem cells (ASCs) from adipose tissue. After continuous subculture and cryopreservation, ASCs still have the potential for multidirectional differentiation. They can be implanted in the human body to promote bone repair after induction *in vitro*, solve the problems of scarce sources and large damage, and are expected to be used in the treatment of bone defects and non-union fractures. However, the diversity of its differentiation lineage and the lack of bone formation potential limit its current applications in bone disease. Here, we concluded the current applications of ASCs in bone repair, especially with the combination and use of physical and biological methods. ASCs alone have been proved to contribute to the repair of bone damage *in vivo* and *in vitro*. Attaching to bone scaffolds or adding bioactive molecules can enhance the formation of the bone matrix. Moreover, we further evaluated the efficiency of ASC-committed differentiation in the bone in conditions of cell experiments, animal models, and clinical trials. The results show that ASCs in combination with synthetic bone grafts and biomaterials may affect the regeneration, augmentation, and vascularization of bone defects on bone healing. The specific conclusion of different materials applied with ASCs may vary. It has been confirmed to benefit osteogenesis by regulating osteogenic signaling pathways and gene transduction. Exosomes secreted by ASCs also play an important role in osteogenesis. This review will illustrate the understanding of scientists and clinicians of the enormous promise of ASCs’ current applications and future development in bone repair and regeneration, and provide an incentive for superior employment of such strategies.

## 1 Introduction

Bone is a complex tissue with unique characteristics such as repair potential and regeneration ability (Apostu et al., 2019), which is carried out by osteoblasts, osteoclasts, osteocytes (Hutchings et al., 2020; Salhotra et al., 2020; Donsante et al., 2021), and nearby functional systems such as the vasculature system (Filipowska et al., 2017), nerve, and humoral regulation (Yang et al., 2021). If the process is inadequate, it will cause a series of clinical complications like non-union defects demanding artificial intervention, especially for some conditions such as congenital malformations, complex trauma, degenerative disease, and tumor resection, which usually require abundant bone tissue for reconstruction. This is a challenge for both patients and doctors, which will lead to the extension of treatment, affect the patient’s physical and mental health, and constitute a major social and economic burden (Schlickewei et al., 2019; Sheen and Garla, 2021).

The standard methods widely used in clinical practice to stimulate or enhance bone regeneration include distraction osteogenesis, bone transport, and bone transplantation. Among them, bone graft substitutes gained more and more attention for their two important characteristics: 1) osteoconductivity, the internal relocation of mesenchymal cells, osteoblasts, osteoclasts, and additional vascular system offered by scaffolds, and 2) osteoinductivity, which stimulates different cell lineages to differentiate into osteoblast lineage, and they should allow rapid angiogenesis without causing any immune rejection or disease spread (Dimitriou et al., 2011; Emara et al., 2015; Holzapfel et al., 2017; Ghassemi et al., 2018; Zhang et al., 2019a). Bone graft substitutes are composed of scaffolds and selected cells that can adhere and differentiate into bone. Based on scaffolds to fix and protect cells, a cell source and additional growth factor can be added to contribute to osteogenesis, that is, bone tissue engineering (BTE) (Perić Kačarević et al., 2020).

In 1991, the term “mesenchymal stem cells” (MSCs) was introduced to designate cells in the “mesoderm” from which bone, cartilage, fat, and other tissues are derived (Marquez-Curtis et al., 2015). Among these cells, bone marrow–derived mesenchymal stem cells (BMSCs) and ASCs have become more comprehensive and in-depth research subjects (Sousa et al., 2014). Compared with BMSCs, it is easier to obtain higher yields of ASCs from the subcutaneous area through minimally invasive and painless surgery. Moreover, ASCs can maintain their phenotype longer in culture, present greater proliferation capacity, and may be more suitable for allogeneic transplantation than BMSCs (Gu et al., 2018). ASCs can be induced into osteogenic lineage by bioactive molecules, and their secretions, especially exosomes or EVs (extracellular vesicles carrying proteins, RNA, DNA, and lipid molecules), are related to fracture healing (Mende et al., 2021). Careful contrast of ASC-EVs, BMSC-EVs, and synovium-derived MSC (sMSC)-EVs show the highest efficiency in osteogenesis *in vivo* and *in vitro* of ASC-EVs (Li et al., 2021). Moreover, ASC-EVs overexpress angiogenic factors, which have an important impact on angiogenesis (Gorgun et al., 2021; Pomatto et al., 2021). It is worth noting that the exosomes of ASCs can promote BMSC migration more than those of BMSCs (Li et al., 2021). This provides another angle and possibility for ASCs to be used in regeneration medicine. The regulation of osteogenic differentiation involves a complex network and several signaling pathways: among them are BMP-, wnt-, and Notch-signaling (Lough et al., 2016; Lowery and Rosen, 2018; Shafaei and Kalarestaghi, 2020). The Wnt-signaling pathway is the crux because it acts as a regulator between ASC lineages, which guides ASCs from adipogenic or chondrogenic lineages to osteogenic differentiation by increasing Runt-related transcription factor-2 (Runx2) and osteoblast-specific transcription factor (Osterix) (Senarath-Yapa et al., 2014). In recent years, these pathways have been continuously improved, and a variety of regulatory molecules have been found and proved in preclinical trials, which will be described in detail below. It is precisely because of the proliferation capacity and differentiation potential of ASCs, as well as the release of paracrine-signaling factors and cell-free EVs that ASCs are widely used in tissue regeneration and human disease (Marquez-Curtis et al., 2015; Shukla et al., 2020). In particular, several bioactive molecules [such as growth factors, reactive oxygen species (ROS), and miRNA] can be secreted through continuous exosome release, which can effectively regulate the surrounding microenvironment (Storti et al., 2019). They can promote cell proliferation, migration, and angiogenesis; suppress cell apoptosis and inflammation; as well as reduce oxidative stress and involve immune regulation (Cai et al., 2020; Park et al., 2021). Compared with MSCs from other sources, ASCs also show the advantages of extensive sources and significant osteogenic effects.

In this article, PubMed and Elsevier databases are used, with ASCs and bone regeneration, bone defect, and fracture as keywords, and the date is limited to 5 years. Here, the preparation and acquisition of ASCs, the cellular, preclinical and clinical practice in bone repair and regeneration, as well as the latest progress of related research, will be comprehensively reviewed, and the underlying influence on its application and development of new materials and technology will be discussed in the following.

## 2 The preparation of ASCs

In order to better apply ASCs for bone regeneration, it is particularly important to obtain sufficient and high-quality cells. Current methods for harvesting adipose tissues include syringes, liposuction, and direct excision (Chen et al., 2016). ASCs for experiment and treatment can be isolated from subcutaneous adipose tissue of the abdomen, thigh, and arm (Si et al., 2019). Methods of isolation and culture of ASCs follow standard protocol; briefly, it involves washing with phosphate-buffered saline (PBS), digestion of fat aspirates with 0.075% collagenase, and then cultured with 10% fetal bovine serum (FBS) and 1% antibiotics at 37°C and 5% CO_2_^33^. The more important thing is the identification and analysis of ASCs by flow cytometry analysis of cell surface markers. The International Society for Cell Therapy (ISCT) and the International Federation of Fat Therapy and Science (IFATS) have specified three minimum standards for the definition of ASCs: 1) cells must be plastic adherent; 2) they must express CD73, CD90, and CD105, but not CD14, CD11b, CD45, CD19, CD79, and HLA-DR; 3) they must have the potential to differentiate into preadipocytes, chondrocytes, and osteoblasts (Palumbo et al., 2018). According to the properties of different cells obtained, we can use targeted detection methods and establish corresponding animal models, such as 3-(4,5)-dimethylthiazole(-z-y1)-3,5-diphenyltriazolam (MTT) assay and wound model establishment for fibroblasts (Zhou et al., 2019), as well as functional assessment of re-innervation for peripheral nerve regeneration (Zhou et al., 2020; Nakajima et al., 2021). However, the standard definition of harvesting and processing technology has not yet been established. We need more extensive research to develop and standardize it, which will greatly contribute to the application of ASCs in regenerative medicine.

## 3 Molecular mechanisms regulating ASC osteogenesis

The majority of studies have demonstrated the molecular mechanism of bone formation of MSCs, especially BMSCs, while data on the mechanism of osteogenic differentiation of ASC are scarce. A variety of transcription factors can regulate osteogenic differentiation, including Runx2 and ALP (alkaline phosphatase), which are detectable osteogenic markers. The confirmed osteogenic molecular mechanism of ASC will be described and concluded below.

### 3.1 Classic osteogenic differentiation pathways

**FIGURE 1:**
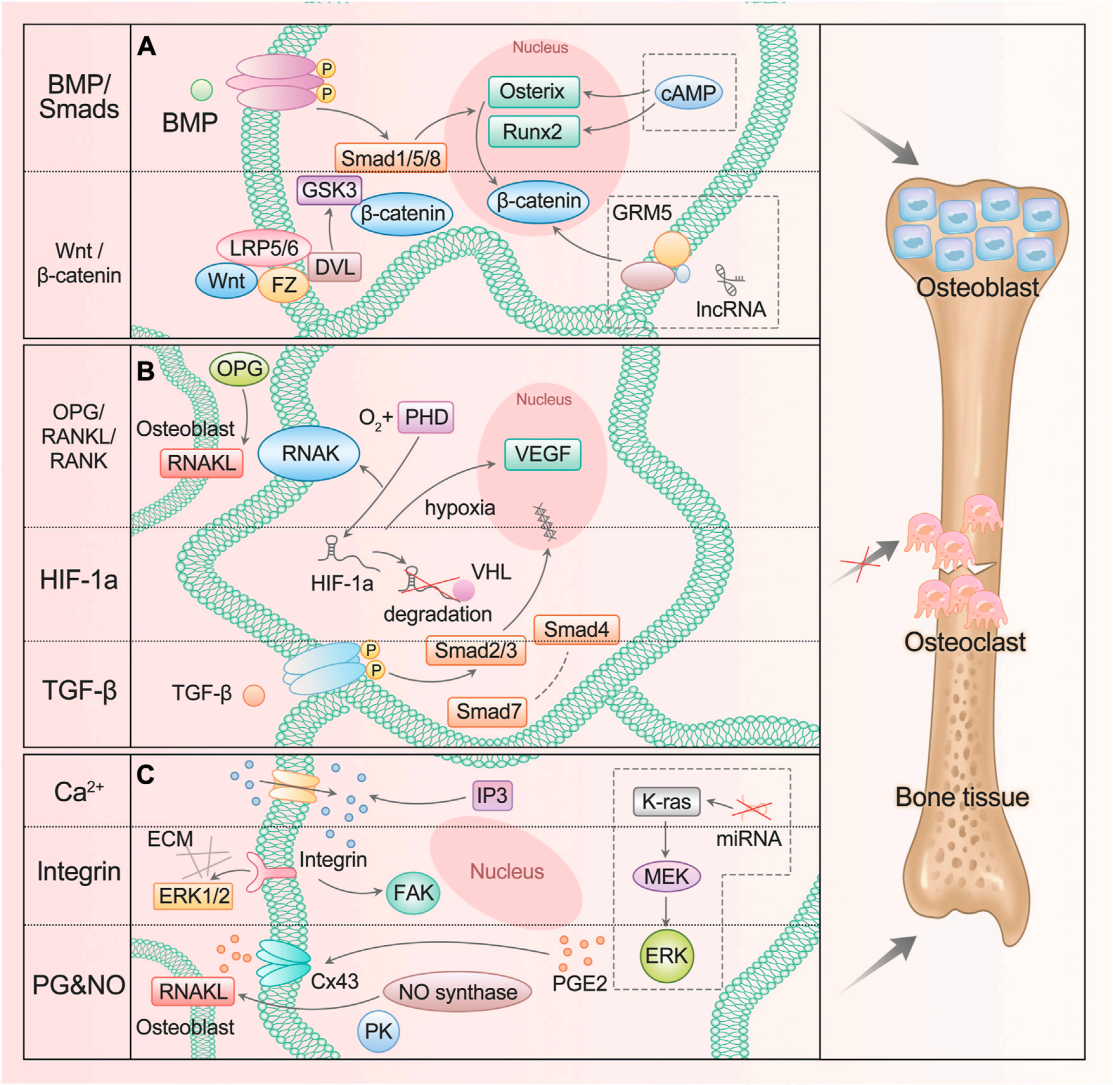
ASC mediates diverse signals to participate in osteogenesis. **(A)** BMP/Smads and Wnt/GSK-3β/β-Catenin pathways promote ASC differentiation into osteoblast. The lncRNA participates in the regulation of the Wnt/β-Catenin signaling pathway, and cAMP can act on Runx2 and Osterix genes. **(B)** OPG/RANKL/RANK, HIF-1a, and TGF-β pathways inhibit ASC differentiation into osteoclast. **(C)** Ca^2+^, integrin, PG, and NO signaling participate in bone resorption and formation. The miRNA enhances the activation of the K-ras/MEK/ERK pathway, which contributes to bone proliferation.

In fact, some classical osteogenic pathways of BMSCs are also found in ASCs and are regulated by many substances, such as Wnt/glycogen synthase kinase (GSK-3β)/β-Catenin axis (Li et al., 2020a) and BMP/Smads signal (Xie et al., 2017), which promote the differentiation into osteoblasts (Figure 1A). When the Wnt pathway is activated, Wnt protein binds to frizzled receptors (Fz) and further binds to helper receptor low-density lipoprotein receptor–associated protein 5/6 (LRP5/6) to form a functional junction receptor complex. FZ can further act on the cytoplasmic dishevelled protein (DVL), activate the DVL in the cytoplasm, and then inhibit the activity of GSK-3β. This antagonizes the phosphorylation and degradation of β-Catenin by GSK-3β and makes the content of β-Catenin in the cytoplasm accumulate and move to the nucleus (Etheridge et al., 2004; Yavropoulou and Yovos, 2007; Zeng et al., 2008). After binding with the BMP receptor, BMP activates the receptor and further phosphorylates the cytoplasmic signal molecule Smad1/5/8. These phosphorylated Smad1/5/8 molecules bind to Smad4 to form a complex and enter the nucleus (Miyazono et al., 2010; Katagiri and Watabe, 2016). β-Catenin and Smads protein in the nucleus interact with the transcription factors Runx2 and Osterix to affect the transcription of osteogenesis-related genes (Katagiri and Watabe, 2016; Ma et al., 2020).

Added to that are the transforming growth factor β (TGF-β) pathway and osteoprotegerin (OPG)/nuclear factor κ B receptor-activating factor ligand (RANKL)/nuclear factor κ B receptor-activating factor (RANK) pathway: they can inhibit the differentiation into osteoclasts and promote osteogenesis (Chen et al., 2012; Chen et al., 2018) (Figure 1B). TGF-β activates Smad2/3 after binding with the receptor. The activated Smads protein forms a complex with Smad4 and then translocates to the nucleus. Smad7 can destroy the activated Smad2/3 and form a complex with Smad4 (Chen et al., 2012). OPG competitively binds to RANKL as a decoy receptor; inhibits the interaction between RANKL and RANK; and regulates the differentiation, proliferation, and apoptosis of osteoclasts (Hofbauer and Schoppet, 2004).

These signaling pathways have been studied and summarized, but there is no comprehensive research and report on ASCs, and there are few related preclinical experiments. However, it is undeniable that the classical osteogenic differentiation pathway is still the best target for the regulation of ASCs. Because they are sound and reliable enough, many activators and inhibitors of corresponding signals have been produced in the market, which is helpful for further experiments. Understanding and mastering different pathways opens a door for the targeted regulation of osteogenic differentiation of ASC.

### 3.2 Hypoxia/hypoxia-inducible factor pathway

The hypoxia-inducible factor (HIF-1a) pathway is involved in the process of ASC osteogenesis (Figure 1B). Under normoxic conditions, the oxygen-dependent degradation domain (ODD) on the HIF-1a oxygen-sensing element is hydroxylated by active prolyl hydroxylase (PHD) and then combined with VHL protein to enter the proteasome for degradation. In hypoxia or hypoxic environment, the activity of PHD disappears or decreases, resulting in the accumulation and transport of HIF-1a to cells, which plays a role by activating HIF-sensitive target genes such as VEGF. As one of the most direct target genes of HIF-1a, VEGF regulates angiogenesis and bone formation through signal transduction (Lee et al., 2004; Liu and Holmes, 2021). It was also found that the HIF-1a pathway can regulate the expression of OPG/RANKL in osteoblasts and affect the differentiation function of osteoclasts (Jin et al., 2015a).

### 3.3 Signals related to mechanical stimuli

Bones respond to mechanical stimuli and activate corresponding signaling pathways including calcium ions, integrins, prostaglandins (PG), and nitric oxide (NO) (Huang and Ogawa, 2010) (Figure 1C). The level of inositol 1,4,5-triphosphate (IP3) promotes the release of calcium ions and increases its cytoplasmic concentration, then activates Wnt pathway and triggers BMP-2 (Ren et al., 2021). Focal adhesion kinase (FAK) is an intracellular component of the integrin signaling pathway, which eliminates osteogenic response when inactivated. ERK also plays a crucial role, which is an important way to connect mechanical transduction and osteogenic differentiation (Shuaib et al., 2019). Shear stress can promote the release of intracellular PGE2 from osteoblasts and induce Cx43 translocation to the membrane surface, making it a new portal to release PGE2 in response to mechanical strain. PGE2 enhances the signal by activating PKA and cAMP-dependent ion channels, resulting in an increase in cAMP and participating in osteogenic differentiation (Lisowska et al., 2018). In addition, binding PGE2 to its receptor can improve bone healing and regeneration by increasing the expression of BMP-2 and RANKL. NO promotes angiogenesis, thus regulating the differentiation of ASC (Kang et al., 2020). NO synthase works synergistically with RANKL, and mitogen-activated protein kinase (PK) participates in it to promote bone remodeling.

### 3.4 Other signal transductions

Evidently, great progress has been made in the study of new signaling pathways and related regulatory molecules of ASC osteogenesis. It has been proved that at high intracellular cyclic adenosine monophosphate (cAMP) concentration, early osteogenesis was significantly inhibited. The up-regulation of cAMP level led to the down-regulation of Runx2 and Osterix expressions, while the down-regulation of cAMP content had no significant effect on the expression of Runx2 and Osterix. On the other hand, after treatment with cAMP enhancers, late osteogenesis was significantly stimulated, and bone mineral content and osteocalcin levels increased significantly. In addition, undifferentiated and pre-differentiated ASCs responded to cAMP pathway stimulation differently in osteogenesis (Rumiński et al., 2020). The application of cAMP enhancers or inhibitors to regulate the osteogenic differentiation of ASCs is just around the corner.

Now there is a lot of evidence that the change in microRNA (miRNA) expression level is related to the osteogenic differentiation of ASCs. It has been determined that several miRNAs have potential roles in promoting or inhibiting the osteogenic differentiation of ASCs. For example, the inhibition of miRNA-143 enhances the activation the of K-ras/mitogen-activated protein kinase (MEK)/extracellular signal-regulated kinase (ERK) pathway during osteogenic differentiation (Zhang et al., 2020b).

Studies have shown that hundreds of lncRNAs are differentially expressed during ASC osteogenesis, and LINC00314 is the most statistically significant, which is found to be prominently upregulated in this process, regulating ASC osteogenic differentiation through metabotropic glutamate receptor 5 (GRM5). It is proved that GRM5 regulates the Wnt/β-Catenin signaling pathway and thus promotes osteogenic differentiation of ASCs (Shi et al., 2020).

**FIGURE 2:**
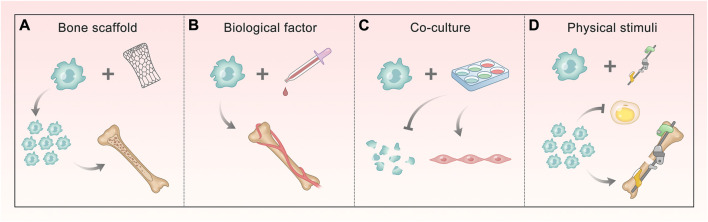
Methods of promoting ASC osteogenesis involving different signals. **(A, B)** Bone scaffold and biological factors involve BMP/Smads, Wnt/GSK-3β/β-Catenin, OPG/RANKL/RANK, and HIF-1a pathways. The former makes ASCs proliferate and form mineralized nodules, and the latter ensures ASCs mediate bone matrix formation and enhance vascular ingrowth. **(C)** Co-culture involves HIF-1a and TGF-β pathway, making ASCs decrease apoptosis and differentiate into vascular smooth muscle cells (VSMCs). **(D)** Physical stimuli correlate with Ca^2+^, integrin, PG, and NO signaling, which do ASCs a great favor in proliferating and attaching, enhancing osteogenesis and reducing the differentiation into fat.

In recent years, the small-molecule substances that regulate ASC osteogenesis are far more than those mentioned above, and the pathways involved are various and complex. We can find that most of them that have been proved to contribute to the regulation of osteogenesis target the classical osteogenic differentiation pathways or osteogenic markers. This illustrates that the discovery of multiple osteogenic pathways is beneficial to exploring the role of more regulatory factors, and also provides a direction for further application (Figure 2). More detailed and comprehensive conclusions are expected to fully understand the regulation network.

## 4 Various ways to promote ASC osteogenesis at the cellular level

**FIGURE 3:**
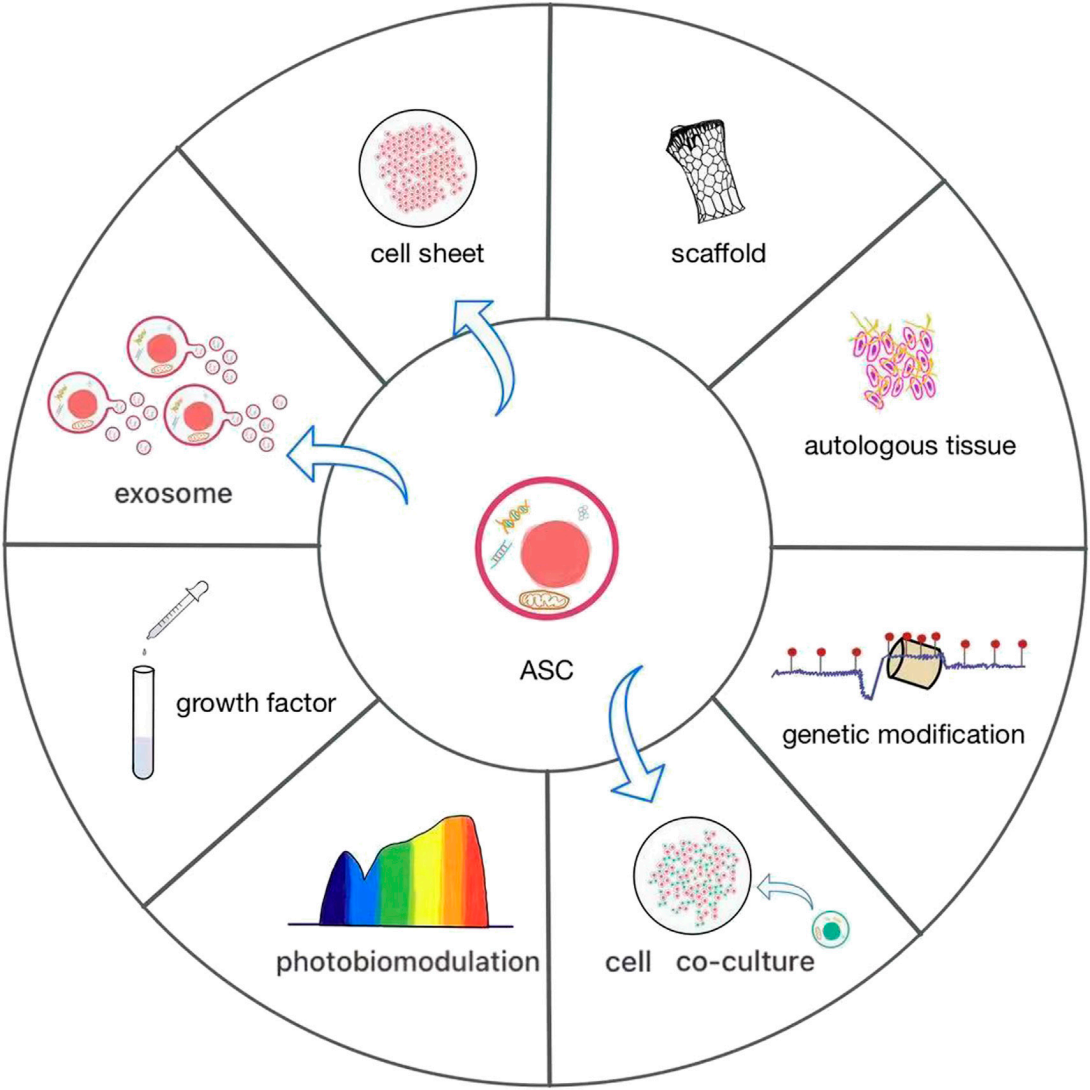
The current various applications of ASCs. ASCs have rarely been used alone, often as cell sheets or combined with scaffolds to promote osteogenesis. It is effective to add autologous tissue and growth factors or construct co-culture systems in ASCs. They are increasingly widely used biological methods, which contribute to the growth and differentiation of ASCs. Photobiomodulation and genetic modification are novel and worthwhile efforts to give an impetus to the osteogenic differentiation of ASCs. Direct application of exosomes of ASCs can also promote bone formation.

Because the osteogenic efficiency of ASCs alone does not satisfy the demand for treatment, they are often combined with physical and biological means to promote bone formation (Figure 3). The bone scaffold is one of the most commonly used methods, which can provide attachment sites for ASC osteogenesis. Different materials can also stimulate the characteristics of ASCs. Biological factors have an impact on the growth and directional differentiation of cells themselves. Using more of existing technologies, we can explore the solution to promote ASC osteogenesis from many aspects through physical stimulation. In addition, bone repair cannot lack good vascularization, which requires us to take a measure to promote osteogenesis from this level–co-culture system. This section will describe and expand this from the above key parts.

### 4.1 Bone scaffolds

Since ASCs contain therapeutic properties including differentiation capability into a variety of cell lineage *in vitro* as well as having immunomodulatory (Cai et al., 2020), osteoinductive, and anti-inflammatory features (Kruger et al., 2018; Yu et al., 2019), they can be used for bone repair and regeneration in cases of non-union fracture or bone defects. The current method widely used in bone transplantation is the combination of scaffolds and ASC sheets to improve bone induction and enhance bone remodeling (Hutchings et al., 2020; Wang et al., 2021a). BTE also contains another key element, that is, bioactive factors (Storti et al., 2019). Ideal scaffold materials must be biodegradable and biocompatible, and have strong osteoinductive properties (Wubneh et al., 2018; Sherman et al., 2019). ASCs have used a variety of organic or inorganic scaffolds for BTE so far, which can be divided into non-metal and metal. Non-metal scaffolds include acellular matrix, ceramic [such as hydroxyapatite (HAP) and coralline-derived HAP], synthetic polymers, and hybrid scaffolds [such as polycaprolactone (PCL) and copolymer polylactic acid-glycolic acid (PLGA)], as well as natural polymers (such as collagen), while metal scaffolds contain metal and alloy materials [such as titanium (Ti) and titanium dioxide (TiO_2_)] (Zhang et al., 2020a). Distinct scaffolds have different advantages and disadvantages. The natural extracellular matrix (ECM) has been widely used as a support of ASCs. However, owing to the poor mechanical behavior and unpredictable biodegradability of natural ECM, some people believe that hydrogels are the most promising alternative materials for their excellent swelling properties and similarity with soft tissues (Huang et al., 2017). The combination of biopolymer and bioceramic can simulate the chemical composition of natural bone ECM, particularly HAP has excellent biocompatibility with various cells and tissues (Rahman et al., 2020). However, the interfacial compatibility between them is poor (Shuai et al., 2020), and the vulnerability of porous bioceramics substitutes cannot match the toughness of bone (Wang et al., 2018a). Synthetic polymers have the advantages of customized and predictable structure and properties, but lack biological activity. Natural polymers usually contain biological functional molecules to ensure biological activity, bionic surface, and natural modification. Their main disadvantages are immunogenic response, uncontrollable degradation rate, and weak mechanical strength (Donnaloja et al., 2020). Natural and synthetic polymer–based materials can be hybridized through 3D (three-dimensional) printing to combine respective advantages and can be created according to the specific needs of patients (Zhang et al., 2019b). The potential of metals and alloys as scaffolds has also been greatly exploited. Porous 3D structure based on TiO_2_ is proposed as scaffold material with high biocompatibility and bone conductivity in large bone defects (Ahn et al., 2018). In addition, it is reported that silver nanoparticles used in scaffolds show excellent antibacterial properties and can reduce the cytotoxicity of ASCs (Calabrese et al., 2021). Notably, the development of nanomaterials and 3D printing technology makes the application of scaffolds more convenient and diversified (Bodnárová et al., 2019; Singh et al., 2020; J Hill et al., 2019). In addition, people are beginning to consider the applicability of scaffolds from the way they are used. Many scaffolds need to be manufactured and placed in bone repair sites; nevertheless, hydrogels are promising materials owing to their injectable properties.

Scaffold-free allogeneic ASCs have been shown to adhere to defects and promote histological healing in rabbit models (Oshima et al., 2019). Research finds that ASCs can adhere closely to the scaffold, proliferate, and form high-density bone tissue (Wang et al., 2021a). Compared with ASC sheets alone, the expression levels of osteogenic markers ALP, osteocalcin, and osteopontin (OPN) increased significantly (Wang et al., 2021b). Another animal experiment showed that the combination of ASCs improved the gap and surrounding ossification of the scaffold compared with the scaffold alone (Lee et al., 2021). ASCs also display the ability to accelerate bone formation after using biological scaffolds (Liu et al., 2021).

### 4.2 Biological factors

Bone regeneration involves the interaction between ASCs and biological factors. The concentration of bioactive factors and the tendency related to the differentiation medium will affect the osteogenic potential of ASCs (Dubey et al., 2018). Stimulating factors could be added to increase the osteogenic potential, proliferation, vascularization, migration, and differentiation of progenitor cells as a supplement to osteogenesis *in vitro* (Xu et al., 2020). BMP is the main bone growth factor group and has been applied in clinical practice despite the contrasting results (May et al., 2019). For example, BMP-2 is an effective osteoinductive growth factor, which is proven in many preclinical studies (Olthof et al., 2019). Retinoic acid was shown to increase the effect of BMP-2 on osteogenic differentiation of human ASCs (Cruz et al., 2019). In addition, BMP-7 demonstrated the capability to promote ASCs to complete bone regeneration in experiments (Kim et al., 2018). Plasma is a rich source of growth factors; several studies used platelet-rich plasma to increase the osteogenic potential of ASCs (Scioli et al., 2017), which was confirmed in the rat skull defect model (Tajima et al., 2018). But in recent years, more studies are focused on platelet-rich plasma which is conducive to the cartilage differentiation of ASCs (Barlian et al., 2018; Rosadi et al., 2019; Barlian et al., 2020). Simvastatin can also enhance osteogenic differentiation, but not in the hyaluronic acid microenvironment (Wu et al., 2021). In addition, exendin-4 (a glucagon-like peptide 1 receptor agonist) promotes bone differentiation and repair while inhibiting adipose differentiation *in vitro* (Deng et al., 2019). Moreover, it is proved that parathyroid hormone (PTH1-34) can phosphorylate sik2, upregulate RANKL, and downregulate SOST, thereby upregulating Wnt4 to promote the osteogenesis of ASCs (An et al., 2019). Glucagon-like peptide-1 (GLP-1) has been demonstrated to promote osteogenic differentiation of human ASCs through the Wnt/GSK-3β/β-Catenin pathway (Li et al., 2020a). Starting from improving ASC growth conditions and regulating osteogenic pathways, the use of biological factors may provide a new insight and attempt for bone regeneration with ASCs.

### 4.3 Physical stimulation

On the other hand, physical stimulation also plays an important role in the osteogenesis of ASCs. There is evidence that the potential of using graphene-cellulose (G-C) scaffolds to enhance cell biological activity through electrical stimulation supports bone induction or reconstruction, which can enhance the proliferation and osteogenic differentiation of ASCs (Li et al., 2020b). One experiment has found that argon plasma modification on the surface of nanocomposite polyurethane scaffolds could have the same effect as before (Griffin et al., 2019). Other studies confirmed that Ti and TiO_2_ surfaces have a positive effect on the osteogenesis of ASCs as well (Malec et al., 2016; Zanicotti et al., 2018; Cowden et al., 2019). It may help increase the osteogenic efficiency of ASCs and translate into clinical practice. Due to the low bone regeneration efficiency of undifferentiated ASCs, 3D culture conditions can be used to mimic the natural stem cell niche, which may increase the osteogenic commitment of ASCs (Rumiński et al., 2019). PCL scaffold is widely considered a suitable MSC delivery system, and is used as a 3D culture environment to promote the osteogenic differentiation of ASCs (Rumiński et al., 2018). G-C paper can be assembled into the 3D structure by alginate lamination as well, which is helpful for ASC culture and osteogenesis (Li et al., 2019).

At present, the exploitation and experiment of novel materials, appropriate photoelectric stimulation, and 3D conditional culture are the main directions of physical stimulation to promote ASC osteogenic differentiation, which helps ASCs play a role in bone defect from another dimension and supports the application of scaffold.

### 4.4 Co-culture system

Bone defects and fractures are often accompanied by vascular destruction; the interaction of angiogenesis and osteogenesis participates in the formation of bone tissue, which is supposed to allow an effective vascularization process to repair bone defects (Diomede et al., 2020). Compared with 2D (two-dimensional) culture, it was proved that the arrangement of ASCs into spheres could make them undergo dual spontaneous osteogenesis and angiogenesis (Gorkun et al., 2021). In order to improve vascularization and osteogenesis in tissue engineering, another promising method is the combination of endothelial cells and ASCs. Studies have shown that co-culturing ASCs and human umbilical vein endothelial cells (HUVECs) can stimulate proliferation, cell survival, osteogenesis, and angiogenesis, especially in 50%: 50% co-culture (Mutschall et al., 2020). In addition, other studies have shown that hypoxia stress accelerates ASC differentiation into VSMC, which may be explained the up-regulation of mettl3 and paracrine factors (Lin et al., 2020). However, some experimental results show that hypoxic pretreatment can reduce the osteogenic differentiation of ASCs by the addition of VEGF (Hwang et al., 2020).

Finally, choosing a younger donor may be beneficial for osteogenesis, and 3D culture is also expected to be used (Payr et al., 2020). All kinds of measures provide many options for ASCs to better differentiate into bone, so it can be more conducive to bone repair and regeneration in different situations. In addition to the impact of diverse single methods on ASC differentiation, maybe we should also explore whether simultaneously using distinct techniques still has a positive effect.

## 5 Distinct animal models of ASCs contributing to bone repair have been established based on cell experiments

Because it is impossible to adequately simulate the complexity of the *in vivo* environment or predict the clinical efficacy *in vitro*, there is a need to prove the role of ASCs in bone regeneration with the help of animal models. Rodents and mammals are widely used and important animal models of ASCs, such as rats, mice, rabbits, dogs, and pigs. According to their different characteristics, they can be divided into large animals and small animals. Rodent models cannot fully mimic human bone regeneration for many reasons, including the lack of cortical remodeling and the fact that growth arrest occurs much later than in other mammals. Similarly, the lower body weight in the small animal models is obviously inconsistent with the biomechanical conditions of human bone load. When exploring the function of ASCs in large bone defects, we are required to use large animal models. In addition, the response process of the immune system between large animal models and humans is more homologous when using bone allografts. Based on the characteristics of various animal models, the outcome of ASCs in boosting bone regeneration will be described separately (Table 1).

**TABLE 1:** Relevant animal experiments in recent years.

Animal model	Operation method	ASC source	Time frame	Bone healing outcomes	Bone injury	Ref.
Rats	ASC sheets	Rats	4 weeks	Bone healing was achieved, and the osteogenic-induced ASC sheets promoted bone repair	Femoral defect	Yoshida et al. (2019)
Mice	ASCs combined with llp2a alendronate	Mice	42 days	The cotreatment of ASC and llp2a-Ale resulted in higher callus volume fraction, higher mineralization of the callus, and higher bone strength	Femoral fracture	Yao et al. (2016)
Mice	ASC sheet with coral scaffold	Rabbits	8 weeks	The combination of coral scaffold and ASC sheets significantly improved bone formation	No damage	Wang et al. (2021a)
Rats	ASCs with PtNPs	Human	4 weeks	The mineralization of chondrocytes is significant, and the fracture is almost healed	Tibial fracture	Chen et al. (2022)
14Rats	Low-power laser irradiation combined with ASCs	Human	16 weeks	Implanted ASDCs and LPLI worked synergistically to increase bone formation	Skull defect	Wang et al. (2018b)
Rats	PBM and DBM with seeded ASCs	Human	8 weeks	Bone formation, remodeling, and consolidation were improved	Femoral defect	Gazor et al. (2021)
Rats	rAd/BMP-2 transduced ASCs	Human	8 weeks	rAd/BMP-2 expedited bone regeneration	Parietal bone defect	Park et al. (2017)
Rats	Circrna-vgll3 overexpression ASCs	Rats	8 weeks	New bone formation by upregulating bone mineral density is significantly enhanced	Critical size bone defect	Zhang et al. (2021)
Dogs	ASC injection and 3D printing PCL/tricalcium phosphate (TCP) coated with bone demineralized and decellularized ECM	Dogs	8 weeks	Ossification was more abundant	Mandibular defect	Lee et al. (2021)
Rabbits	ASCs combined with fibrin glue scaffold	Rabbits	56 days	Cortical bone reconstruction was significant and new cortical bone bridges formed	Mandibular defect	Mehrabani et al. (2018)
Rabbits	ASC sheet–EPC complexes	Rabbits	8 weeks	Newly mineralized tissue formed	Skull defect	Wang et al. (2021c)
Rabbits	DCS complex and PLL modified CHA	Rabbits	12 weeks	A large number of well-arranged layered bones formed	Radius defect	Zhang et al. (2019c)

### 5.1 Small animal models

Rats and mice are mature animal models of ASCs in bone repair research, which has confirmed that ASCs promote osteogenesis through different patterns. It has been indicated that even uncultured and minimally processed ASCs can also improve the pathological bone healing secondary to radiation (Lynn et al., 2020). This tells us that ASCs have unlimited prospects for bone repair with some improvements.

#### 5.1.1 Physical and chemical stimulation

As expected, it is proved that ASCs and osteogenic-induced ASC sheets significantly promoted the bone healing rate of femoral bone defects in rats (Yoshida et al., 2019). The production of cell sheets is conducive to improving the osteogenic efficiency of ASCs. The content of osteocalcin (OCN)–positive osteoblasts in osteogenic-induced ASC sheets increased after transplantation, but there was no significant difference in CT images and bone mineral density in bone defect areas between ASCs. In addition, there is evidence that ASCs combined with llp2a alendronate (a bone-seeking compound) can accelerate fracture healing in a mouse closed-fracture model, improve callus formation, and promote angiogenesis (Yao et al., 2016). The animal experiments of ASCs integrated with various kinds of scaffolds to repair bone also obtained satisfying outcomes. The combination of coral scaffold and ASC sheet was implanted under the skin of nude mice and differentiated into osteoblasts in the ectopic part, which significantly improved bone formation (Wang et al., 2021a). ASCs have also derived a new application strategy in bone repair. One study constructed and pre-vascularized a mesoporous bioactive glass scaffold using endothelium-induced ASCs and combined it with osteogenic ASCs (Du et al., 2018). This enables time-phased sequential use of ASCs on scaffolds and provides a novel direction for further research. A new study found that supercritical carbon dioxide acellular bone matrix seeded with ASCs accelerated the formation of new bone in a rat model. It is shown that PCL scaffolds with the transient release of hyperoxia enhance ASC-mediated skull regeneration in mice (Farris et al., 2022). These findings provided biological clues for the application of scaffolds (Liu et al., 2021). Nanomaterials have made new progress in ASCs contributing to bone formation. A study combined platinum nanoparticles (PtNPs) with ASCs to treat tibial fractures in rats, confirming that PtNPs promote the osteogenic differentiation of ASCs *in vitro*, accelerate fracture healing, and have no significant impact on the differentiation of ASCs into chondrocytes and adipocytes (Chen et al., 2022). It is worth affirming that there is no statistical difference between PtNPs and the control group when evaluating the results, which indicates that PtNPs may play a role by affecting some osteogenic signal pathways, laying the foundation for further research.

Some studies have proved that ASCs combined with physical stimulation therapy is effective. In the rat bone defect with biparietal critical-sized bone defects (6 mm in size), the use of photobiomodulation (PBM) combined with ASCs wrapped in methacrylic acid gelatin hydrogel improved bone regeneration *in vivo* (Calis et al., 2021). Moreover, low-power laser irradiation combined with ASCs significantly enhanced the process of bone healing in the rat model of skull defect (Wang et al., 2018b). It is confirmed that PBM and demineralized bone matrix (DBM) combined with ASCs can increase new bone formation in the rat model of osteoporosis (Gazor et al., 2021). However, the femoral defect area of the ASC-seeded group is larger than that of PBM and DBM without seeded ASCs, which is puzzling. Despite positive outcomes of photobiological stimulation in the treatment of regenerated bone with low-intensity laser, no standardized parameters have been established to obtain repeatable results (Da Silva et al., 2021). The optimal quantity of ASCs that can affect bone repair has not been verified, and these experiments did not demonstrate the more common fracture models in clinical practice.

#### 5.1.2 Biomaterials

In addition to considering the non-bioactive means, the ready-made bioactive factors or tissues may also be helpful for ASC osteogenesis. It has been proved that the combination of concentrated growth factor (CGF) and ASC sheets can promote bone regeneration in rat skull defect models, and the optimal concentration of CGF extract is 20% (Hu et al., 2021). The co-culture system also played a corresponding role. A collagen-based hydrogel 3D scaffold experiment demonstrated that the co-cultured ASC and osteoblasts showed enhanced bone formation in the rat skull defect model compared with the ASC single culture group (Kim et al., 2020). Also, the vascularization and bone formation of the osteogenic matrix composed of ASCs and HUVECs were successfully demonstrated through the angiogenesis induced by arteriovenous loop (AVL) surgery in the rat model (Winkler et al., 2021). Exploiting different co-culture systems and studying the paracrine effect in these systems are the next steps.

#### 5.1.3 Signaling pathway

Some studies target the signal pathways of osteogenic differentiation and successfully helped ASC improve the efficiency of bone repair. A relevant experiment managed to promote the osteogenic differentiation and enhance bone regeneration of ASCs by the use of adenovirus expressing BMP-2 and porous PLGA scaffold compared with the ASCs group without transduction in the rat model of the parietal bone defect (Park et al., 2017). There are studies indicating that the use of Dickkopf-1 (DKK1, a Wnt antagonist) neutralizing antibody can improve the early osteogenic differentiation of human ASCs *in vitro* (Negri et al., 2021). Early studies have shown that systemic anti-DKK1 therapy is capable of improving the healing process in rodent models of long bone fractures (Jin et al., 2015b).

From another study, it is known that the major epigenetic mechanisms involved in DNA methylation, histone modification, and RNA interference (RNAi) represent one of the determinants of ASC differentiation (Chen et al., 2019). In previous research, it is found that the combination of mir-135(a miRNA related to osteogenesis) overexpression ASC and poly (sebacoyl diglyceride) (PSeD) scaffold significantly promoted new bone formation in critical size skull defects of rats (Xie et al., 2016). A recent study proved that circRNA-vgll3 (circular RNAs originating from the vgll3) significantly enhances new bone formation by upregulating bone mineral density, bone and tissue volume, and trabecular number in the critical size defect model of rats (Zhang et al., 2021). In addition, it was recently reported that changing the expression of exosome miRNA can promote the osteogenesis of human ASCs, while there are no adequate and clear animal experiments to prove it (Yang et al., 2019). However, this does not prevent us from making progress in the regulation of ASC osteogenic differentiation. In evidence, these findings could provide potential therapeutic targets for bone regeneration medicine in the future. Encouragingly, a new study developed a CRISPR-Bid system, which uses three mechanisms: transcriptional activation, histone acetylation, and DNA methylation to inhibit fat-induced genes and activate cartilage-induced genes, conducive to repairing bone defects in osteoporotic rats (Truong et al., 2022).

### 5.2 Large animal models

Compared with small animal models, large animal studies are relatively few, which is due to different animal sources and experimental tasks. In dogs receiving ASC injection and 3D printing PCL/tricalcium phosphate (TCP) coated with bone demineralized and decellularized ECM, mandibular ossification in and around the scaffold hole was improved (Lee et al., 2021). There was no sign of infection and immune rejection, but this may also be due to the short follow-up time after surgery (8 weeks). In a 2018 trial, ASC-related fibrin glue scaffolds were used to improve mandibular defects in rabbits, and the effect was not significantly different from that of autologous bone transplantation (Mehrabani et al., 2018).

The idea of induced differential and co-culture ASC has also made some progress in large animal experiments. Osteogenic ASC sheet–endothelial progenitor cell (EPC) complexes bi-directionally differentiated and formed dense and well-vascularized new bone tissue 8 weeks after implantation, which repaired the skull defects of rabbits (Wang et al., 2021c). The vascularized tissue-engineered bone constructed by the double cell-sheet (DCS) complex and poly-L-lysine (PLL)–modified coral hydroxyapatite (CHA) was proved to have the potential to repair large radius bone defects in rabbits (Zhang et al., 2019c). DCS provides a large number of osteoblasts and vascular endothelial cells, and PLL effectively promotes the proliferation and differentiation of ADSC. A study using ASC sheets and autologous platelet-rich fibrin (PRF) to repair peri implant defects in canine mandible showed that PRF significantly enhanced the proliferation and osteogenic potential of canine ASCs, which provided a new strategy for BTE (Ding et al., 2019). This only increases the amount of bone around the implant, and the effect of large bone defects is unknown. However, for the majority of bone defects in large animal experiments, scaffolds are still needed to attach cells for their growth. It is the key to obtaining good results in the experiment to clarify the characteristics, advantages, and disadvantages of various scaffold materials and select them.

### 5.3 Summary of animal experiments

Evidently, there are more studies on small animals and also more ways, and there have been examples of combining targeted molecular pathways with animal models. Large-animal research still stays at the level of combination of ASCs with scaffold or co-culture with autologous tissue, lacking further technical improvement and extension of molecular experiments. In addition to the differences between small animals and large animals discussed above, the reasons also include that the conclusions obtained from small-animal experiments are not very perfect and clear. There is a void of sufficient samples and exploration of specific mechanisms, and the ASC transplantation group is not significantly superior in some evaluations (such as imaging). It is shown that ADSCs can survive for up to 4 months after injection into rat tissue and have good long-term survival and differentiation ability, but there is no similar evidence in large-animal models (Muñoz et al., 2018). In addition, these preclinical trials have rarely explored the chronic inflammatory response after ASC transplantation. Specific paracrine effects and cytokines that really work also need to be further studied.

## 6 The promises and challenges of ASCs in clinic

### 6.1 Present situation of ASCs for bone repair treatment

Due to the possible clinical benefits of ASCs, many relevant clinical trials have been launched, but only a few of them involve bone healing or bone regeneration. Among the corresponding experiments that have been started, only about one-third of projects were indicated as completed (Le et al., 2021). Some clinical trials adopt ASC transplantation alone, others use scaffolds to combine with ASCs, and some of them add additional growth factors. Most of the trials that have published the results are for the treatment of craniomaxillofacial bone injuries or defects, a few are about non-union fractures, and the clinical effect is generally meaningful. A clinical trial in 2015 confirmed that autologous ASCs without any scaffold can be fully differentiated into 3D osteogenic implants, which can safely promote long bone non-union fractures (Dufrane et al., 2015). There are no acute and long-term side effects within 4 years, and the cure rate reaches 3/6. According to the outcomes of several clinical trials for the treatment of skull defects, the transplantation of autologous human ASCs did not have major adverse reactions, the formation of focal bone trabeculae was good, and a small number of patients achieved excellent clinical results, but there were problems of graft loosening, infection, and tumor recurrence (Torres-Guzman et al., 2022). However, a six-year follow-up of cranioplasty based on ASCs, β-TCP, and supporting mesh illustrated unsatisfactory results, that is, they are not superior to conventional cranioplasty (Thesleff et al., 2017). There were no adverse events within 3 years after implantation, indicating the security of cell therapy, although some people were concerned about the choice of tumor cell cloning. The anatomical structure and function of bone have been restored, but the incidence of bone non-union has increased due to complications. Some clinical trials have been completed, the relevant information has not been released and the reason is vague. More recent clinical trials on bone have been applied to joint and disc disease. Two clinical trials in 2018 and 2019, respectively, showed that intra-articular injection of ASCs can improve knee arthritis without adverse reactions, which may have the potential to prevent disease progression (Song et al., 2018; Freitag et al., 2019). Intradiscal injection of ASCs and hyaluronic acid in the treatment of chronic discogenic low back pain was proved to be safe and tolerable, but the effect is not quite significant (Kumar et al., 2017).

We can see that not only are the means of ASC application less diverse, but also the bone sites that can be repaired by ASC transplantation are relatively limited. Some patients who enter clinical trials will inevitably have the outcome of infection and tumor recurrence due to complications such as diabetes and tumors. Though a mass of preclinical studies illustrated that ASCs have great value for bone repair and regeneration, there are still some difficulties and barriers in related clinical trials.

### 6.2 Difficulties of ASCs in clinical bone regeneration

Many reasons lead to the limitation and concern of the clinical application. First and foremost, ASCs may be contaminated during the preparation process, thus making it obstruct osteogenesis. Various clinical trials adopt distinct methods to separate and extract ASCs, which will also make a difference; a unified standardized method may be needed in the future. Purification of stem/progenitor cells by cell sorting technology can effectively avoid this problem. However, due to its complexity, it is a challenge for the regulation of clinical transformation. Perhaps another effective but simpler solution is to pharmacologically target the signaling pathways expressed in the unpurified stromal cell population that may inhibit osteogenic differentiation or activate transcription factors that regulate osteogenesis, thereby improving osteogenic efficiency and enhancing ossification. For example, one study used the chemical activator TM-25659 of TAZ (a transcriptional coactivator with PDZ-binding motif demonstrated to modulate osteogenic differentiation of MSCs) to pharmacologically activate the osteogenic differentiation and damage the fat differentiation of ASC, finally promoting bone formation in mice (Zhu et al., 2018). ASCs treated with TM-25659 produced more bone-like structures and collagen deposition, and new bone formation increased significantly. However, the delivery strategy and dosage of corresponding drugs need to be optimized and discussed. When added to the human body, its metabolic process and possible adverse reactions also need to be further studied.

In addition, the application of ASC therapy often requires the addition of extra growth factors to maintain the proliferation of cells. Although BMP-2 has been approved by the US FDA for clinical use in 2003 as a stimulant of bone formation in ASCs, the amount of BMP-2 required to induce bone formation in large grafts exceeds the physiological level without significant improvement in efficiency and clear long-term impact (Emara et al., 2015; Rindone et al., 2019). Similar to pharmacological activators, the addition of exogenous cytokines needs to clarify the efficacy, dosage, and adverse reactions in order to achieve the optimal effect. This requires us to study the mechanism of ASC osteogenesis more thoroughly, including the amount and regulation of related factors, so as to better react to all kinds of situations when ASCs are applied to the human body.

At the same time, the biological dynamic characteristics of ASC are not equal to those of normal bone. Some scholars worry that ASC differentiation *in vitro* may lead to tumor formation, which requires that the time of ASC amplification *in vitro* should be as short as possible. It is not uncommon for ASC to become loose after transplantation, so it is necessary to ensure that the grafts can form a shape of appropriate size and are conducive to fixation in practical application. Patients who had their bones removed due to tumors were also more likely to discontinue the trial due to tumor recurrence. Some patients developed a late infection in the late follow-up period. Although it may be due to the patient’s complication factors such as diabetes and smoking, it cannot be ruled out that the transplantation caused the chronic inflammatory reaction. Except for the causes of tumor recurrence and infection, the tough issue is insufficient ossification. This leads to the failure to form sufficiently hard and dense bone tissue after transplantation. Therefore, the clinical benefits of ASC transplantation need to be further demonstrated. In recent years, the effect of ASCs in the treatment of intervertebral discs and joints has been affirmed. We know that the differentiation of ASCs into cartilage is better than that in osteogenesis, but we urgently need to understand the reasons and strategies. The safety of ASC transplantation has been verified, and the short-term effect is also in line with expectations. However, more trials are needed to prove how to preferably deal with the graft and clarify the clinical indications.

### 6.3 Further solutions

Based on the above shortcomings, we may be able to try to promote the treatment of ASC starting from three aspects: First and foremost, more attention needs to be given to the mechanism of ASC differentiation into bone. On this basis, we can make a lot of progress. It is reported that paracrine cytokines, exosomes, and other active substances are the main factors for ASCs to exert their biological effects (Praveen Kumar et al., 2019); adipose-derived acellular derivatives appeared as an alternative to the treatment of ASCs recently (Cai et al., 2020). In addition, many experiments are limited to the combination of therapeutic biological agents and ASCs, but do not explore the benefits of using biological agents alone. We need to make more attempts to obtain and establish a standard formula of bioactive factors for the treatment of ASC. The 3D sphere culture could give a valuable experimental setting for the study of ADSC osteogenesis regulation because it provides effective osteogenesis induction in the standard differentiation medium without growth factor. In addition, the application of natural medicine compounds (such as ginger, garlic, turmeric, and green tea) in bone diseases has attracted much attention. Due to their harmlessness, they could be used to increase the therapeutic effect of ASCs (Bose et al., 2021). In addition, perhaps genetic modification and transduction will be one of the mainstream methods for the remedy of ASCs with clear mechanisms and mature technology (Bougioukli et al., 2018).

Second, a continuous attempt should be made to optimize the structural, chemical, and surface properties of rigid scaffolds to enhance bone regeneration. We need more discovery and testing of new materials to promote the development of osteogenic scaffolds. When conducting tests, care should also be taken to exclude laboratory animals and experimental subjects from being affected by systemic diseases, such as osteoporosis and diabetes, that may affect bone repair. Last but not least, the enhancement of osteoblast helper cell activity should also be stressed on: it may be another door for us to assist the osteogenesis of ASCs to achieve clinical transformation.

## 7 Conclusion

This overview summarizes the progress of ASCs in bone regeneration and repair in recent years, including *in vitro*, *in vivo*, and clinical trials. The corresponding improvements and shortcomings are also mentioned, and the solutions for reference are put forward. Bone defects seriously threaten the prognosis of some diseases, such as malignant tumors and serious infections. With the growth of the population life, the ability to self-repair of the human body decreases with age; particularly the quality of life of elderly patients with a non-union fracture is increasingly poor. ASC treatment brings new hope for large bone defects and non-union fractures due to its proliferation and differentiation potential, low immunogenicity, and minimal injury, which inspires those suffering patients. Researchers are also actively involved in the research and have obtained many positive results. Bone scaffold, physical and chemical stimulation, bioactive materials, co-culture system, gene regulation, and other methods combined with ASCs have been proved to significantly enhance bone formation in preclinical trials, and relevant clinical trials have also shown relatively positive results on the whole. However, we are still faced with problems of inefficient differentiation of ASCs and difficulties in clinical transformation. We hope that the emergence of more strategies and experiments can greatly promote the osteogenesis of ASC and facilitate its clinical application. Although there are some barriers to be crossed, we should also believe in the unlimited potential and good development trend of ASC treatment. With the new materials and technologies expanding, the applications of ASC will be more diverse and variable.

## Glossary

ALP: alkaline phosphatase
ASC: adipose-derived mesenchymal stem cell
AVL: arteriovenous loop
BMSC: bone marrow–derived mesenchymal stem cell
BTE: bone tissue engineering
cAMP: cyclic adenosine monophosphate
CGF: concentrated growth factor
CHA: coral hydroxyapatite
DCS: double cell-sheet
DKK1: dickkopf-1
DVL: cytoplasmic dishevelled protein
ECM: extracellular matrix
EPC: endothelial progenitor cell
ERK: extracellular signal regulated kinase
EV: extracellular vesicles
FAK: focal adhesion kinase
FBS: fetal bovine serum
Fz: frizzled receptors
G-C: graphene–cellulose
GLP-1: glucagon-like peptide-1
GRM5: metabotropic glutamate receptor 5
GSK-3β: glycogen synthase kinase
HAP: hydroxyapatite
HIF-1a: hypoxia-inducible factor
HUVEC: human umbilical vein endothelial cell
IFATS: International Federation of Fat Therapy and Science
IP3: inositol 1,4,5-triphosphate
ISCT: International Society for Cell Therapy
LncRNA: long noncoding RNA
LRP5/6: low-density lipoprotein receptor-associated protein 5/6
MEK: mitogen-activated protein kinase
miRNA: microRNA
MSC: mesenchymal stromal cell
MTT: 3-(4,5)-dimethylthiazole(-z-y1)-3,5-diphenyltriazolam
NO: nitric oxide
OCN: osteocalcin
ODD: oxygen-dependent degradation domain
OPG: osteoprotegerin
OPN: osteopontin
Osterix: osteoblast-specific transcription factor
PBM: photobiomodulation
PBS: phosphate-buffered saline
PCL: polycaprolactone
PG: prostaglandins
PHD: prolyl hydroxylase
PK: protein kinase
PLGA: polylactic acid–glycolic acid
PLL: poly-L-lysine
PRF: platelet-rich fibrin
PTH: parathyroid hormone
PtNP: platinum nanoparticle
RANK: nuclear factor κ B receptor-activating factor
RANKL: nuclear factor κ B receptor-activating factor ligand
RNAi: RNA interference
ROS: reactive oxygen species
Runx2: runt-related transcription factor-2
sMSC: synovium-derived MSC
TCP: tricalcium phosphate
TGF-β: transforming growth factor β
Ti: titanium
TiO_2_: titanium dioxide
VSMC: vascular smooth muscle cell
2D: two-dimensional
3D: three-dimensional
